# Ecosystem Effects of Variant Rabbit Hemorrhagic Disease Virus, Iberian Peninsula

**DOI:** 10.3201/eid2012.140517

**Published:** 2014-12

**Authors:** Miguel Delibes-Mateos, Catarina Ferreira, Francisco Carro, Marco A. Escudero, Christian Gortázar

**Affiliations:** Instituto de Investigación en Recursos Cinegéticos, a collaborative agency of the Consejo Superior de Investigaciones Científicas, Universidad de Castilla-La Mancha, and Junta de Comunidades de Castilla-La Mancha, Ciudad Real, Spain (M. Delibes-Mateos, C. Gortázar);; Trent University, Peterborough, Ontario, Canada (C. Ferreira);; Estación Biológica de Doñana, Seville, Spain (F. Carro);; Ebronatura, Zaragoza, Spain (M.A. Escudero)

**Keywords:** Rabbit hemorrhagic disease virus, calicivirus, European rabbit, *Oryctolagus cuniculus*, Iberian lynx, ecosystem, Iberian Peninsula, viruses

**To** **the** **Editor**: In this investigation, we found evidence for the apparent effects that a new variant of the rabbit hemorrhagic disease virus (RHDV) is having on native wild European rabbit (*Oryctolagus cuniculus*) populations on the Iberian Peninsula, and how this virus could threaten the conservation of endangered predators.

Historically, European rabbits were extremely abundant on the Iberian Peninsula, which is in their native range. However, during the 20th century, the number of rabbits on the peninsula has declined >90%, mainly because of diseases (*1*). The first notable crisis among rabbits occurred during the 1950s concurrent with the arrival of myxomatosis among rabbit populations, which caused mortality rates of ≈90% (*1*), as registered in other regions. During the late 1980s, a calicivirus, RHDV, caused infections that made a strong impact on rabbit populations, causing initial mortality rates of 55%–75% in Iberia (*1*). Since their initial outbreaks, both diseases have become enzootic, and related mortality rates have decreased, in part because of increased host resistance, although the infections still play a major role in the dynamics of rabbit populations (*2*).

In 2011, a new variant of RHDV, which appears to be closely related to an isolate originating in France that was described in 2010 (*3*), caused high mortality rates in some rabbit farms in Spain (*4*) and was also identified in an experimental wild rabbit plot in northern Spain (*5*). Since 2012, the new variant of RHDV has been detected in most rabbit farms in Spain (*6*), and in several wild populations distributed across Spain and Portugal (*7*), suggesting that it has rapidly spread throughout the Iberian Peninsula. This variant affects both of the wild rabbit subspecies (*O. cuniculus cuniculus* and *O. c. algirus*), and unlike the classical form of RHDV, it kills rabbits as young as 11 days of age and rabbits that have been vaccinated against classic RHDV (*6*,*7*). This scenario has raised concern for the survival of wild rabbit populations and its predators in this region.

Data regarding rabbit trends seem to sustain this concern. For example, a long-term monitoring program in Aragón in northern Spain shows a notable decline in rabbit numbers during 2013 in populations that showed both long-term increasing and decreasing trends over the monitoring period (Figure, panels A, B, respectively). A similar trend has been observed in the main areas inhabited by the highly endangered Iberian lynx (*Lynx pardinus*). The lynx relies on rabbits for survival, because they represent >85% of the lynx’s diet (*9*). For instance, in Coto del Rey, the area within Doñana National Park in southern Spain that traditionally held the highest rabbit densities and therefore represents the core of Iberian lynx populations in this national park, there was a decline in rabbits of >80% during 2012–2013 (Figure, panel C). Similar declines have been detected in low-density rabbit populations surveyed within Doñana National Park (Figure, panel C). Rabbit numbers have also been progressively dropping in the proximity of the Yeguas River in Andújar and Cardeña Natural Parks in southern Spain, where the largest Iberian lynx population currently lives: rabbit density was >3.5 rabbits/hectare in 2010 and <1 rabbit/hectare in 2013, a decline of ≈75% (*10*). In accordance with field surveys, hunters throughout Iberia claim that the number of rabbits harvested this season has decreased dramatically, pointing to a 70%–80% decline compared to the previous hunting season in some estates (A. Linares, pers. comm.).

**Figure:**
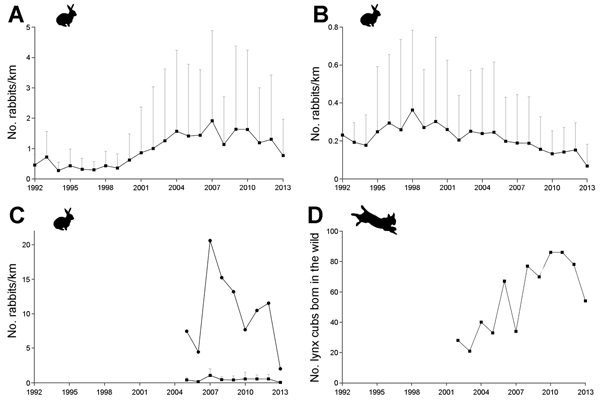
Trends in rabbit abundance (number of rabbits/km) in Aragón and Doñana National Park, northern and southern Spain, respectively, and in the number of Iberian lynx cubs born in the wild in Spain. A) Average rabbit abundance (+SD) of populations showing long-term increasing trend over the whole sampling period (n = 18) in Aragón (*8*); B) average rabbit abundance (+SD) of populations showing long-term decreasing trend over the whole sampling period (n = 25) in Aragón (*8*); C) rabbit abundance over the study period in Coto del Rey (circles), which is likely the main area for rabbits and lynxes within Doñana National Park; and average rabbit abundance (+SD) over the study period of 7 low-density populations (squares) within Doñana National Park (see details about methods in http://www-rbd.ebd.csic.es/Seguimiento/mediobiologico/conejo/pnd/ProtocoloCensoConejosPND.pdf); and D) total number of lynx cubs born in the wild during 2002–2013 in Spain (data available at http://www.lifelince.org and http://www.juntandeandalucia.es).

The European rabbit is a multifunctional keystone species of the Iberian Mediterranean ecosystem, where it serves as prey for >30 predatory animals, alters plant species composition and vegetation structure through grazing and seed dispersal, its excrement and urine have an effect on soil fertility and plant growth and provide feeding resources for invertebrates, and its burrows provide shelter for different species (*9*). Therefore, the decline in rabbit numbers could have potential cascading effects on ecosystem function. In fact, some of these effects may already be apparent on rabbit-reliant animals. On one hand, the sharp reduction in rabbit numbers observed in 2013 in the main lynx distribution area has been accompanied by a notable decrease in the number of lynx cubs born in the wild (Figure, panel D). On the other hand, the number of lynxes killed on roads doubled in 2013 (n = 14) in relation to 2012 (n = 7), and this has been linked to increased lynx displacements related to rabbit scarcity potentially associated with the impact of the new variant of RHDV (http://www.juntadeandalucia.es).

The situation described exemplifies how emerging diseases can affect biodiversity conservation. It also highlights the importance of using wildlife monitoring schemes as detection tools for monitoring the impact of stochastic factors, such as the variant RHDV, on wildlife populations. Urgent management actions, designed within an Iberian rabbit conservation strategy that relies on a multidisciplinary framework, are needed to ensure the conservation of this keystone member of the Iberian Peninsula ecosystem and that of rabbit-reliant predators.
